# Intracellular Streptococcal Uptake and Persistence: A Potential Cause of Erysipelas Recurrence

**DOI:** 10.3389/fmed.2019.00006

**Published:** 2019-01-29

**Authors:** Fatma Jendoubi, Manfred Rohde, Jörg Christoph Prinz

**Affiliations:** ^1^Department of Dermatology, University Clinics, Ludwig-Maximilian University of Munich, Munich, Germany; ^2^Faculty of Medicine of Tunis, University Tunis El Manar, Tunis, Tunisia; ^3^Central Facility for Microscopy, Helmholtz Centre for Infection Research, Braunschweig, Germany

**Keywords:** erysipelas, cellulitis, relapse, intracellular streptococcal uptake, intracellular streptococcal persistence, risk factors, treatment, penicillin prophylaxis

## Abstract

Erysipelas is a severe streptococcal infection of the skin primarily spreading through the lymphatic vessels. Penicillin is the treatment of choice. The most common complication consists in relapses which occur in up to 40% or more of patients despite appropriate antibiotic treatment. They cause lymphatic damage resulting in irreversible lymphedema and ultimately elephantiasis nostras and lead to major health restrictions and high socio-medical costs. Prevention of relapses is an unmet need, because even long-term prophylactic penicillin application does eventually not reduce the risk of recurrence. In this article we assess risk factors and causes of erysipelas recurrence. A systematic literature search for clinical studies addressing potential causes and measures for prevention of erysipelas recurrence was combined with a review of experimental and clinical data assessing the ability and clinical relevance of streptococci for intracellular uptake and persistence. The literature review found that venous insufficiency, lymphedema, and intertrigo from fungal infections are considered to be major risk factors for recurrence of erysipelas but cannot adequately explain the high recurrence rate. As hitherto unrecognized likely cause of erysipelas relapses we identify the ability of streptococci for intracellular uptake into and persistence within epithelial and endothelial cells and macrophages. This creates intracellular streptococcal reservoirs out of reach of penicillins which do not reach sufficient bactericidal intracellular concentrations. Incomplete streptococcal elimination due to intracellular streptococcal persistence has been observed in various deep tissue infections and is considered as cause of relapsing streptococcal pharyngitis despite proper antibiotic treatment. It may also serves as endogenous infectious source of erysipelas relapses. We conclude that the current antibiotic treatment strategies and elimination of conventional risk factors employed in erysipelas management are insufficient to prevent erysipelas recurrence. The reactivation of streptococcal infection from intracellular reservoirs represents a plausible explanation for the frequent occurrence erysipelas relapses. Prevention of erysipelas relapses therefore demands for novel antibiotic strategies capable of eradicating intracellular streptococcal persistence.

## Introduction

### Definition of Erysipelas

**Figure 1:**
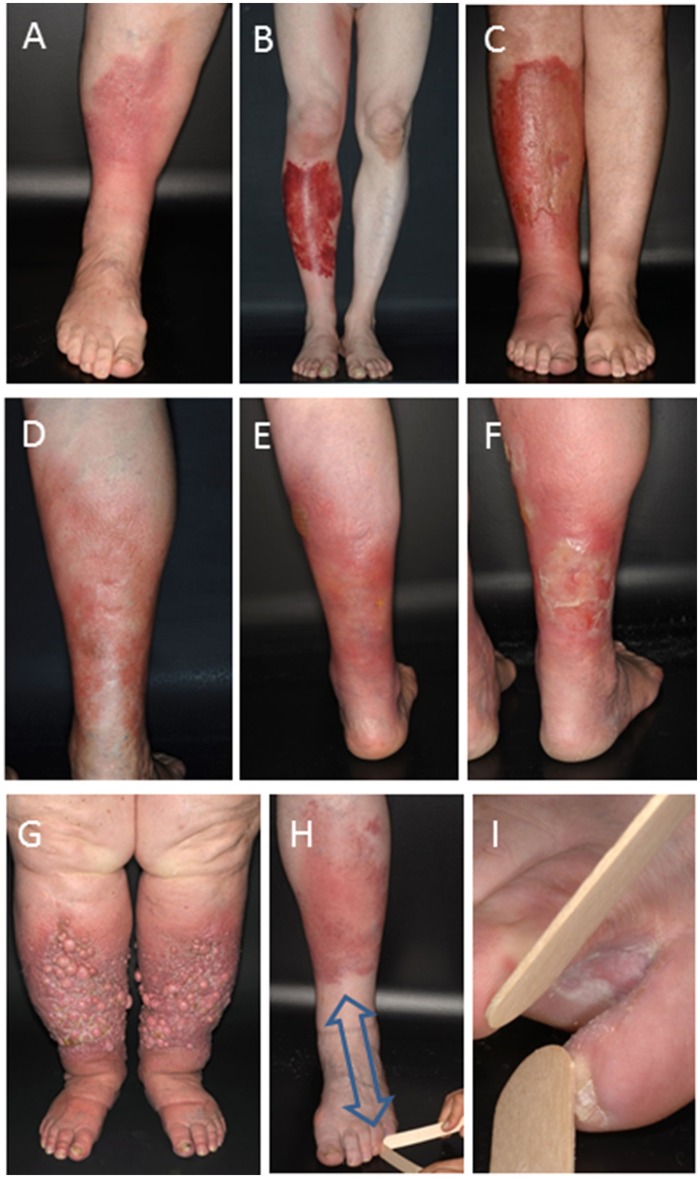
Clinical manifestation of erysipelas and erysipelas relapses. Typical inflammatory **(A)** or hemorrhagic **(B)** erysipelas of the lower leg. **(C)** Increase in lymphedema following several relapses in bullous erysipelas as indicated by the circumferential increase of the left ankle and non-pitting edema of the forefoot. **(D)** First erysipelas episode and two relapses two **(E)** and three **(F)** years after the first episode typically occurring in the same site of a female patient. **(G)** Late-stage excessive lymphedema with thickened fold of skin at the base of the second toe that cannot be lifted (positive Stemmer sign test), dermal fibrosis, and formation of numerous fibrotic nodes from multiple episodes of relapsing erysipelas. **(H,I)** Toe-web inoculation site of a first erysipelas episode of the left lower leg. Note the typical centripetal distance (arrow) between bacterial inoculation site and erysipelas.

Erysipelas is a severe infection of the skin mainly caused by β-hemolytic group A streptococci (*S. pyogenes*, GAS) (1–4). Streptococcal infection in erysipelas primarily affects the lymphatic vessels (5). As already defined in the scriptures of Hippocrates, “έρυσίπελαϛ” (red skin) presents as an acute onset of local inflammation with painful edematous erythema that has a sharp border to the adjacent unaffected skin. Tongue-like or irregular extensions reflect the spreading of infection by way of the lymphatic vessels (Figures 1A–C). Accompanying systemic symptoms are fever, chills, malaise, and laboratory signs of inflammation (6, 7). The terms erysipelas and cellulitis are often used interchangeably. They are commonly seen as manifestations of the same condition, whereby erysipelas is thought to primarily affect the superficial skin layers, i.e., epidermis, dermis, and upper subcutis, while cellulitis is considered a more diffuse skin infection extending from dermis deeper into subcutaneous tissue (8). Clinically, the borderline between erysipelas and cellulitis is blurred and a true differentiation may be difficult. In this review, the terms of erysipelas and cellulitis are considered different designations for the same disease.

Erysipelas is a global health burden. The reported incidence of erysipelas ranges from 19–24 per 10,000 inhabitants in European countries to 24.6 cases per 1,000 patient years depending on the study population analyzed (9–11). In the United States of America, an estimated 14.5 million cases per year cause more than 650,000 admissions and $3.7 billion U.S. dollar in ambulatory care costs (12). In a strict classification, erysipelas is caused by β-hemolytic group A streptococci (*S. pyogenes*, GAS), but also non-group A streptococci. *S. aureus* and gram-negative bacteria have occasionally been implicated in clinical conditions resembling erysipelas and cellulitis (1–3). Streptococcal infection in erysipelas primarily affects the lymphatic vessels (5). The most common site of the infection according to the primary inoculation site is the lower limb, accounting for about 80% of all cases (Figure 1) (13). The knowledge about the natural course of untreated erysipelas is imprecise. Without adequate treatment erysipelas may cause endocarditis, sepsis and streptococcal toxic shock syndrome (STSS). It may further progress to necrotizing fasciitis involving all layers of the skin, myositis, and myonecrosis (12, 14–16). Non-suppurative sequelae are rare, but cutaneous infections with nephritogenic GAS strains predispose patients to post-streptococcal glomerulonephritis. Rheumatic fever is not associated with streptococcal skin infections (17, 18).

Penicillin is considered the treatment of choice as it is inexpensive and *S. pyogenes* has remained susceptible to β-lactam antibiotics despite 60 years of extensive use (19–22). Although it has been used as the primary treatment for streptococcal infection for decades, *S. pyogenes* has never acquired beta-lactamase genes or penicillin binding protein-based resistance to penicillin (20). Macrolide antibiotics represent an alternative, but resistance rate of GAS is increasing (23–25).

### Erysipelas Recurrence: An Unmet Need in Erysipelas Treatment

The most common complication of erysipelas is recurrence with the development of lymphedema. Recurrent episodes of erysipelas occur in up to ~40% of cases and usually affect the same anatomic site (Figures 1C–F) (26, 27). Each recurrent episode of erysipelas causes progressive damage and obliteration of lymphatic vessels (28, 29). This impairs lymphatic drainage and finally results in irreversible lymphedema (Figures 1C,E,F) that might become disabling and has been called “elephantiasis nostras” due to its clinical resemblance of the late stages of lymphedema from lymphatic filariasis (Figure 1G). Elephantiasis represents a dramatic and irreversible condition characterized by deforming lymphedematous swelling and woody fibrosis of the affected anatomic region. Overall, erysipelas relapses are associated with considerable morbidity, social impairment, and health care cost utilization (12, 30).

Long-term low dose prophylactic penicillin is recommended for preventing erysipelas recurrence. Ongoing penicillin prophylaxis prolongs the time to the next episode, although occasionally patients experience relapses during antibiotic prophylaxis (26, 31–33). The protective shield, however, is not sustained after prophylaxis has been discontinued, and the relapse rate again becomes the same as without prophylaxis (26, 34, 35). Accordingly, the issue of preventing erysipelas recurrence remains unsettled. Identifying the causes and developing strategies for preventing relapses therefore represent major unmet medical needs in erysipelas patients.

In this article, we review the mechanisms that have been proposed as explanations for recurrence. Conventional risk factors for relapses are the same as for single episodes (36). They include to the anatomic site, venous insufficiency, lymphedema, previous surgery, continued disruption of the cutaneous barrier facilitating repetitive bacterial entrance, obesity, and other general risk factors (34, 35, 37–42). Allover, however, they do not provide a specific rationale for erysipelas recurrence beyond the risk factors for erysipelas itself. Since penicillin resistance is hardly documented among streptococci, other aspects must therefore be relevant for the high frequency of repetitive erysipelas episodes. Although this is obvious, a recent meta-analysis criticized that only a small number of studies have actually addressed the causes of recurrence (35).

In searching for alternative explanations, we identify intracellular persistence of *S. pyogenes* as potential cause of relapses. Intracellular streptococcal uptake into epithelial and endothelial cells, macrophages, and polymorphnuclear cells creates reservoirs for GAS persistence (43, 44). Because beta lactam antibiotics do not reach sufficient bactericidal intracellular concentration these intracellular reservoirs are likely not eliminated during penicillin treatment. These intracellular reservoirs may serve as dormant infection prone to reactivation. In consequence, we conclude that antibiotic regimens with effective intracellular antibacterial efficacy are required for sustained erysipelas treatment. This demands for alternative treatment strategies and their potential verification in therapeutic antibiotic trials.

## Methods

**Figure 2:**
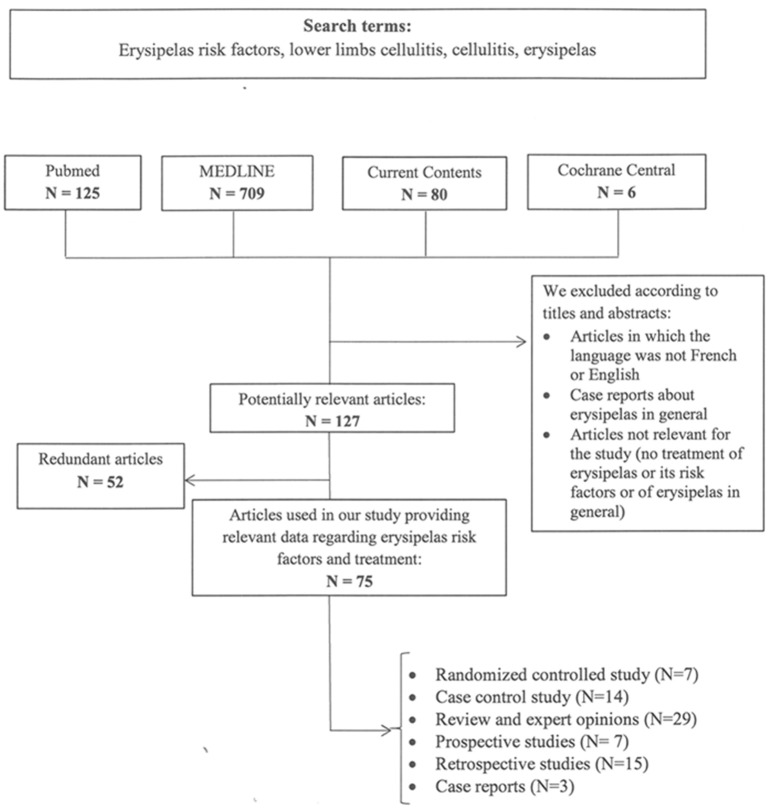
Search terms and flow diagram of literature analysis for recurrence of erysipelas. N designates the number of articles.

**Figure 3:**
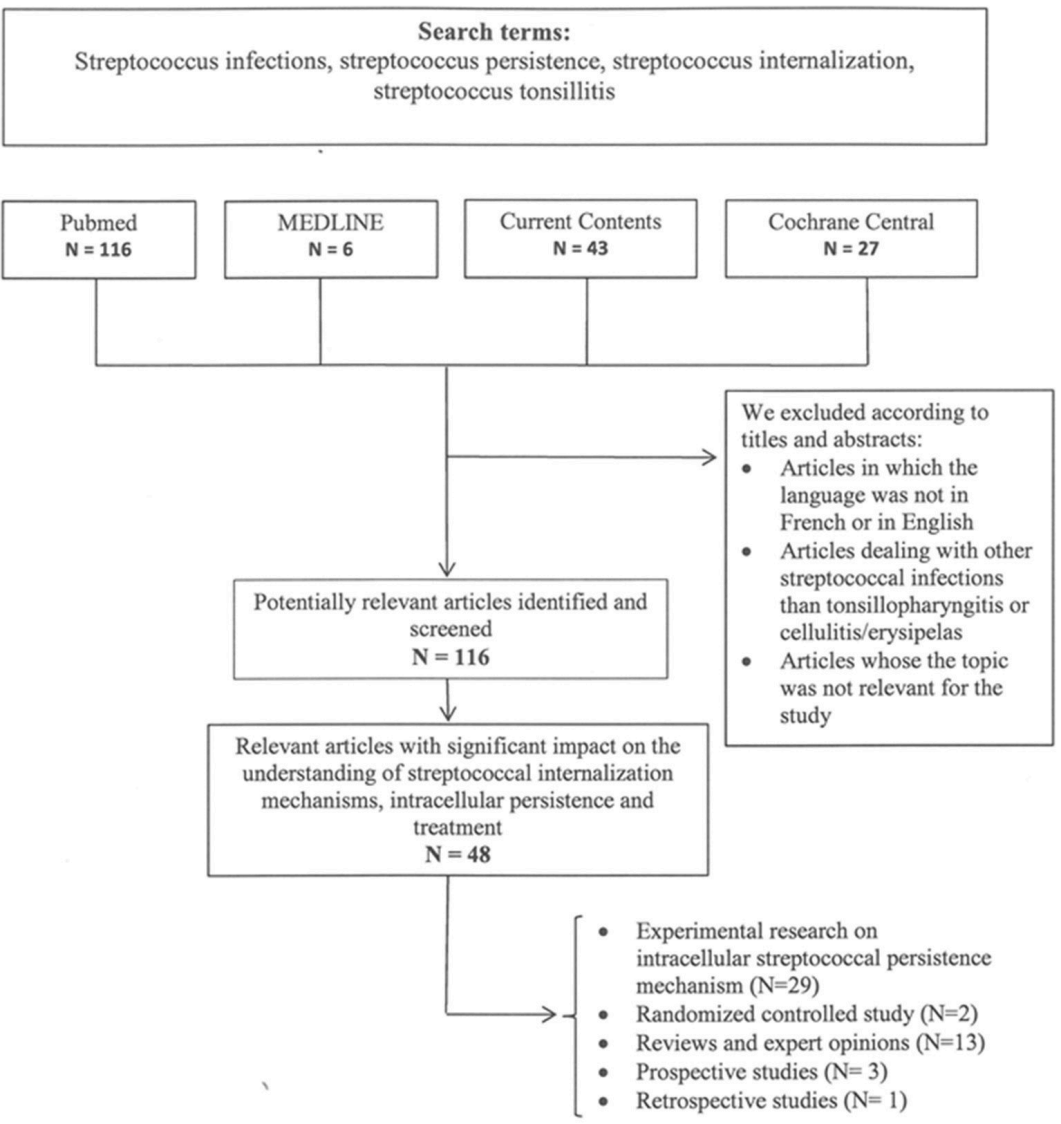
Search terms and flow diagram of literature analysis for intracellular streptococcal uptake and persistence. N designates the number of articles.

Data for this Review were identified by searches of MEDLINE, Current Contents, PubMed, and Cochrane central, and references from relevant articles (Pubmed) addressing erysipelas risk factors and treatment regimens using the search terms “erysipelas risk factors,” “lower limbs cellulitis,” “cellulitis,” “erysipelas,” “streptococcus” or a combination of those terms. Only articles published in English and French until June 2017 were included. We furthermore summarize data on experimental and clinical evidence of intracellular GAS uptake and persistence of GAS infections identified by the search terms “streptococcus infections,” “streptococcus persistence,” “streptococcus internalization,” “streptococcus tonsillitis.” The search strategies and reference assessments are given in Figures 2, 3.

## Conventional Risk Factors of Erysipelas Recurrence

Risk factors for erysipelas recurrence are considered the same as for single episodes (36). They refer to the anatomic site, venous insufficiency, lymphedema, previous surgery, continued disruption of the cutaneous barrier facilitating repetitive bacterial entrance, obesity, and other general risk factors.

### The Anatomic Site

In a population-based cohort study of lower extremity cellulitis, the anatomic site was shown to be the strongest independent predictor of 2-year recurrence. In fact, patients with tibial area cellulitis were five times more likely to experience recurrence than those with cellulitis in the foot or femoral region. Minor traumata to the anterior tibial region and the sparse pretibial subcutaneous tissue were considered as reasons for both pretibial predilection site and erysipelas recurrence (39).

### Venous Insufficiency and Lymphedema

The presence of venous insufficiency and chronic lymphedema were independently associated with erysipelas in many studies and are therefore considered as strong risk factor for recurrent episodes of the lower leg (34, 37, 38, 40, 45–48). Damage to lymphatics leads to ineffective drainage and lymphedema, impairs surface phagocytosis by polymorphonuclear leukocytes, and greatly increases susceptibility to infection (49). Abnormal lymphatic drainage was observed in the unaffected leg of 79–86% of patients who underwent lymphoscintigraphy after a previous episode of cellulitis. Accordingly, patients presenting with leg cellulitis may have pre-existing undiagnosed primary lymphatic abnormalities (28, 29). However, distinguishing venous edema from lymphedema may lack diagnostic sensitivity in particular since chronic venous edema always involves a component of lymphedema (46). Examples for other causes of lymphedema in recurrent erysipelas are radical mastectomy with axillary node dissection or filariasis (50, 51).

Each episode of erysipelas progressively damages lymphatic vessels, increases lymphatic impairment and promotes residual edema (Figures 1C,E,F), creating a vicious cycle predisposing for further erysipelas episodes (52, 53). Severely deranged lymphatic drainage is found in up to 80% of patients with two or more episodes of erysipelas after treatment and resolution of a recurrent episode (54). Thus, edema may be both cause and consequence of the first erysipelas episode, but a distinct causative role for erysipelas recurrence is difficult to prove. Any edema must therefore be considered as risk factor of erysipelas in general and is also associated with recurrence.

### Previous Surgery

Case control studies showed that patients who presented with recurrent erysipelas more frequently have a history of leg surgery (37, 47, 48, 52). Saphenectomy was identified as one of the most prominent risk factor for erysipelas recurrence (52). Twenty-six percent of patients who had undergone radical vulvectomy for vulvar carcinoma with excision of the proximal part of saphenous veins and deep inguinal lymphadenectomy experienced acute leg cellulitis with a recurrence rate of 81%. No risk factor other than the presence of β-hemolytic streptococci at various sites prior to surgery could be identified (55). These findings were confirmed by other studies (56, 57) and explained by the impaired venous and lymphatic drainage following surgery, which had caused bacterial entry sites for colonizing GAS.

### Cutaneous Barrier Defects

Bacterial infections require entrance sites. Defects in skin integrity provide portals of entry for streptococci in erysipelas. Case-control investigations demonstrated that cutaneous barrier disruption was the most highly significant risk factor of erysipelas recurrence (37). In three other case control studies leg wounds were identified as potential risk factors of recurrence (47, 48, 58). Leg ulcers and excoriating skin diseases (eczema, psoriasis, and undefined skin diseases) were also associated with an increased risk for recurrence (37, 47, 48, 58, 59).

Dermatophyte colonization of the skin creates epidermal disruption and fissures for streptococcal inoculation. Tinea pedis is one of the most significant local risk factors of recurrence (34). A European case-control study identified dermatomycosis as an erysipelas risk factor with an odds ratio of 2.4 in univariate analysis (59). This was confirmed by a systemic review and meta-analysis of risk factors of cellulitis of the leg and indicated an association between the presence of toe-web intertrigo (Figure 1I) secondary to tinea pedis and cellulitis (60). Persistent presence of entry sites for bacteria is considered a predisposing factor for recurrent erysipelas, and treatment of tinea is a recommended measure for reducing the risk of cellulitis. Interestingly, the 50% recurrence rate in the antibiotic prophylaxis study by Kremer et al. occurred despite treatment for tinea pedis (61). Tinea treatment may therefore not be sufficient for prophylaxis of recurrence. Moreover, given the ubiquity of tinea pedis in the general population, it was concluded that other factors are involved in the pathogenesis of recurrent erysipelas (28).

### General Risk Factors

Local risk factors appear to play a more significant role in the development of recurrent cellulitis than systemic risk factors. From four general factors (Diabetes mellitus, obesity, smoking, and alcohol intake) identified by a systemic review, only obesity (body mass index >30) was associated with increased risk of recurrence (60). This may be explained by the fact that severe obesity leads to venous and lymphatic impairment as well as a greater risk of fungal infections and thus predisposes for erysipelas in principle.

Malignancies including breast cancer, endometrial cancer, Hodgkin's disease, adenocarcinoma of prostate, and seminoma are other risk factors for streptococcal infections (62). In fact, patients with a history of cancer have 4-fold elevated risk of recurrence compared with patients without any tumor history (39). This may involve venous and lymphatic impairment due to direct tumor effects, radiotherapy or lymphadenectomy (63).

### Conventional Risk Factors: No Sufficient Explanation for Erysipelas Relapses

Overall, the risk factors identified for relapses reflect those for erysipelas in general and do not provide a specific explanation for erysipelas recurrence. Accordingly, a previous episode of cellulitis is the most obvious risk factor for leg cellulitis (37, 47, 59). Curing potential inoculation sites is a principle rationale in erysipelas treatment, and predisposing conditions such as lymphedema or toe-web intertrigo should be effectively treated. However, no robust data are available that these measures can actually affect the relapse rate. In fact, treating tinea pedis does apparently not prevent a second erysipelas episode (61). Further, the protective effect of long-term antibiotic prophylaxis diminishes after discontinuation (26, 34, 35). Thus, the high recurrence rate requires alternative explanations. These may be sought in the biological behavior of streptococci.

## Erysipelas Recurrence: The Role of Intracellular Streptococcal Persistence

### Streptococci and the Ability for Intracellular Uptake and Persistence

While *S. pyogenes* in principle is an extracellular pathogen, certain strains have the ability for intracellular uptake into and persistence within epithelial and endothelial as well as immune cells including macrophage-like cells, polymorphonuclear granulocytes, and dendritic cells (64–75). Intracellular survival seems to be a common streptococcal strategy. Besides Group A streptococci (GAS) also Group C and Group G streptococci are increasingly recognized for their ability to invade epithelial or endothelial cells (76, 77). Following intracellular uptake into viable cells bacteria are protected from the action of β-lactam antibiotics, because penicillins do not penetrate into living cells to a significant extend. Various strategies facilitate intracellular streptococcal uptake and persistence.

### Mechanisms of Intracellular GAS Uptake and Persistence

Efficient intracellular invasion of *S. pyogenes* in non-phagocytic epithelial cells was first suggested by LaPenta et al. using a cell culture infection model in 1994. The ability for intracellular uptake was equal to or even greater than that of classical intracellular pathogens such as *Listeria* or *Salmonella* (65). Thereafter, several studies investigated the mechanism of invasion and survival of streptococcus within throat cells including oropharyngeal keratinocytes to explain treatment failure in pharyngitis (65, 78, 79).

**Figure 4:**
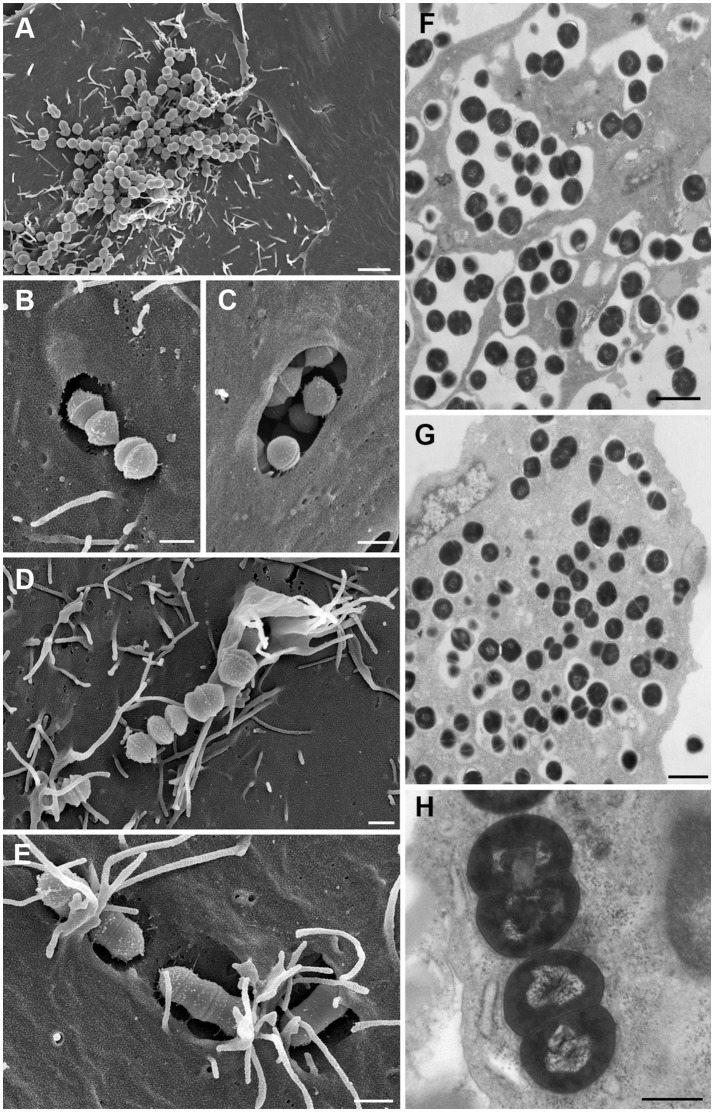
The intracellular life-style of Group A streptococci. **(A)** Adhesion to host cell membrane of HEp-2 cell via adhesins. **(B,C)** Invasion into HEp-2 cells via invasins by co-opting caveolae for forming large invaginations for SfbI-carrying streptococci. **(D)** Invasion through signaling events which trigger cytoskeletal rearrangements like membrane ruffling. **(E)** Adherent streptococci are overgrown by host cell microvilli which fuse to form membrane flaps that subsequently cover streptococci. **(F)** Streptococci residing intracellularly exclusively in membrane-bound phagosome-like compartments after short infection time (cell culture model). **(G)** Release of streptococci out of the membrane-surrounded compartment into the host cell cytoplasm after longer infection time where they replicate and persist (cell culture model). **(H)** *In vivo* mouse model providing evidence of streptococci residing and replicating free in the cytoplasm and supporting the observations generated in cell culture. Bars represent 2 μm in **(A)** 1 μm in **(F)**, and G, 0.5 μm in **(B–E,H)**. All images were taken from infection experiments performed in cell culture models and in a mouse model at the Helmholtz Centre for Infection Research. Experimental procedures were previously described (81, 82). **(A–E)** represent field emission scanning electron microscopic images; Figures (**F–H**) are transmission electron microscopic images of ultrathin sections of resin embedded samples.

Expression of a broad and variable spectrum of adhesins and invasins is one of the hallmarks of streptococci (80). The adhesins accomplish an intimate contact with host extracellular matrix proteins (ECM) such as fibronectin, while invasins trigger the invasion into host cells. Through the repertoire of invasins and adhesins, streptococci have developed numerous mechanisms to internalize and survive in host cells and escape antibiotic treatment and host immune defense (Figures 4A–E) (17, 83–87). M proteins and fibronectin binding proteins (such as sfbI) act as adhesins that mediate attachment to both epithelial and endothelial cells (81, 82).

Two morphologically distinct main invasion mechanisms and different intracellular trafficking routes for streptococci into epithelial and endothelial cells have been described. SfbI and M proteins initiate the process by binding fibronectin which interacts with integrin receptors. Extensive integrin-clustering triggers caveolae aggregation to form large invaginations of the cell membrane that ingest attached streptococci (Figures 4B,C). Alternately, streptococci that engage fibronectin with the M protein may induce focal adhesion complexes which are ingested through a zipper like or membrane ruffling mechanism (Figure 4D) (80). For the caveolae-mediated uptake mechanism GAS prevent fusion with lysosomes and instead reside in caveosomes (82). GAS invading via cytoskeleton rearrangements fuse with lysosomes to form phagolysosomes. Furthermore, in cell culture models GAS can be found residing free in the host cell cytoplasm after longer infection times where they can replicate efficiently (Figures 4F,G) (70, 88, 89). After they have replicated and destroyed the host cells, *S. pyogenes* egress and may infect new cells and may persist in the throat for a long time, releasing any type of streptococcal antigens and causing the permanence of high-antibody titers even in the absence of overt disease. Besides their capacity for invading epithelial and endothelial cells streptococci can escape from the phagosome into the cytoplasm of phagocytic cells where they survive the intracellular killing mechanisms of polymornuclear cells (Figure 4H) (43). Phagocytosis of streptococci alters the apoptosis differentiation program in neutrophils. It up-regulates genes encoding key effectors of apoptosis, and down-regulates receptors critical to innate immune function, potentially resulting in pathogen survival (90). Interaction of certain GAS strains with plasminogen promotes an integrin-mediated attachment and invasion of GAS into keratinocytes, creating a skin-specific reservoir of streptococcal persistence (91).

### Intracellular Streptococcal Persistence in Soft Tissue Infections

In invasive acute soft tissue infections GAS were found within macrophages in biopsies of patients even after prolonged intravenous treatment with antibiotics. The intracellular localization of GAS varied depending on the severity of infection and was most prevalent in newly involved, lesser inflamed tissue with a lower bacterial load, while purely extracellular GAS or a combination of intra- and extracellular GAS dominated in severely inflamed tissue. *In vitro, S. pyogenes* survived intracellularly in macrophages despite therapeutic concentration of penicillin G in culture medium, and antibiotic removal was followed by striking extracellular bacterial growth (92).

The capacity of GAS to invade endothelial cells may facilitate dissemination into surrounding soft tissue. Beyond local invasion, streptococci hiding in the endothelial barrier may also be a source of transient bacteremia (80). *S. dysgalactiae subsp equisimilis* (SDSE) caused recurrent bacteremic infections over several years despite the presence of opsonizing antibodies against the isolate and long-term penicillin treatment. SDSE was found in an aortic aneurysm and the isolate efficiently invaded endothelial cells, thus highlighting the mechanisms of streptococcal persistence in the endothelial lining of vessel walls (77). This may explain why cases of invasive soft tissue infections have been reported in patients without apparent sites of bacterial entry into soft tissue. In line herewith M3-serotypes of GAS are among the most frequent bacteria isolated from patients with invasive tissue disease. These isolates are characterized by a high ability to invade into and persist in endothelial cells. These mechanisms likely contribute to survival, deep tissue tropism, and recurrent spread of invasive GAS (93). Overall, these results imply that streptococci residing intracellularly represent an important reservoir of bacteria that can cause continued infection upon release from their host cells (92).

### Intracellular Streptococcal Persistence Causes Relapses in Recurrent Streptococcal Pharyngitis

The analysis of recurrent streptococcal tonsillitis may help to improve the pathogenetic understanding of the high relapse rate of erysipelas. *S. pyogenes* is the most frequent cause of recurrent bacterial tonsillopharyngitis. A 10-day course of penicillin is the accepted treatment for streptococcal angina (94–96). Similar to erysipelas, treatment with penicillin fails to eradicate streptococcal infection in ~10–30% of patients, even though streptococci remain susceptible *in vitro* to penicillin (97, 98). Uptake into a protected intracellular environment beyond the reach of penicillins may explain treatment failures in streptococcal pharyngitis (66, 68). Intracellular streptococcal reservoirs resisting antibiotic eradication have been reported in recurrent tonsillitis (68, 69). Electron microscopy and immunohistochemistry observed intracellular streptococci in pharyngeal epithelial cells in the majority of patients with tonsillitis and in macrophage-like cells and epithelial cells of asymptomatic GAS carriers (69). After having replicated in and destroying the host cells, *S. pyogenes* egress and may infect new cells and persist in the throat for a long time, releasing streptococcal toxins, and causing permanently elevated antistreptococcal antibody titers even in the absence of overt disease (99). The rate of identical *S. pyogenes* isolates recovered during initial episodes and recurrence of tonsillitis reaches up to 80% as assessed by molecular typing (100), indicating that recurrence is mostly due to reactivation of persistent streptococcal infection in asymptomatic carriers rather than to exogenous reinfection. The ability for intracellular uptake into tonsillar or pharynx epithelium is a particular feature of these streptococcal strains. Unlike strains that were successfully eradicated by antibiotic treatment, they efficiently adhered to and were internalized by Human epithelial type 2 (HEp-2) cells, a cell line originating from a human laryngeal carcinoma (66, 101). Entry into epithelial cells thus provides a robust rationale to explain the persistent pharyngeal colonization as it protects intracellular streptococci from immune effectors and antibiotics which are less membrane-permeable, in particular penicillin (43, 70).

### Intracellular Uptake and Persistence Is a Potential Mechanism for Recurrence of Erysipelas

The findings on the biological behavior of streptococci provide a conclusive rationale for the high recurrence rate of erysipelas that occurs despite appropriate penicillin treatment and long-term antibiotic prophylaxis. They suggest that the ability for intracellular streptococcal uptake and persistence represents an important mechanism responsible for causing erysipelas recurrence. By today, only a few studies have addressed this issue directly. In biopsies from various streptococcal soft tissue infections (myositis, necrotizing fasciitis, and two cases of cellulitis) high amounts of intracellularly residing intact streptococci were observed even after prolonged antibiotic therapy. They had resisted killing by antibiotic treatment presumably due to the intracellular persistence in host cells. The extracellular and intracellular bacterial load in these infections varied upon the severity of infection and tissue involvement. In more than 40% of analyzed cases a purely intracellular bacterial distribution was observed, predominantly in macrophages (92). Antibiotic strategies to prevent recurrent erysipelas episodes should therefore possibly consider the eradication of intracellular streptococcal reservoirs.

## Treatment Strategies for Erysipelas

### Addressing Intracellular Streptococcal Persistence for Preventing Erysipelas Recurrence

It is obvious from clinical trials and practical experience that current antibiotic regimens using β-lactam antibiotics can control acute streptococcal infections in erysipelas but are insufficient in preventing erysipelas recurrence. Strategies addressing common erysipelas risk factors, in particular curing inoculation sites for pathogen entry such as toe-web intertrigo, are necessary for preventing exogenous reinfection but apparently cannot reduce recurrence risk. Together, these observations indicate that both current antibiotic regimens and measures targeting presumed risk factors do not address the actual causes of erysipelas recurrence and need to be reevaluated.

Insights into intracellular GAS uptake and persistence may now provide an intriguing and plausible explanation for prevention failure. Penicillins and cephalosporins poorly penetrate the cell membrane and do not reach sufficient intracellular bactericidal concentrations. Accordingly, intracellular streptococci are protected from β-lactam antibiotics (102). Kaplan et al. observed no degradation of intracellular streptococci after exposure to bactericidal levels of penicillin in a cell culture model while exposure of epithelial cells to either erythromycin or azithromycin could kill intracellular GAS efficiently. Cephalothin and clindamycin were also more effective in killing ingested GAS than was penicillin but less effective than erythromycin or azithromycin (103, 104). Such findings support the hypothesis that the high incidence of erysipelas recurrence and failure of penicillin to prevent erysipelas relapses may indeed be related to intracellular streptococcal uptake and a lack of intracellular penicillin activity (104).

### Current Erysipelas Treatment Guidelines

Although many studies have analyzed the response of erysipelas and cellulitis to various antibiotic regimens, a recent meta-analysis could not define a “best” treatment (105). There is no internationally agreed standard treatment for comparison, and even an appropriate length of antibiotic treatment could not be defined because dosing regimens and outcome measures were heterogeneous between studies (105, 106).

Current guidelines for erysipelas treatment recommend antibiotic therapy according to disease severity without considering potential streptococcal persistence. They propose monotherapy with beta-lactam antibiotics or clindamycin for mild to moderate non-purulent skin and soft tissue infections and advise combination of these antibiotics only for severe cases confirmed by culture and sensitivity tests (22, 107). Levofloxacin, a gyrase inhibitor with enhanced activity against Gram-positive bacteria, may also be used but emergence of resistance is possible (108). Alternatives include linezolid, a first of a new class of oxazolidinone antibiotics with intracellular activity. Linezolid may penetrate phagocytes and non-phagocytic cells. It was as effective as clarithromycin or cefpodoxime proxetil (a third-generation cephalosporin) in the treatment of patients with uncomplicated soft tissue infections and had better outcomes than empiric treatment with oxacillin (109, 110). There was limited evidence that macrolide and streptogramin are slightly better than penicillin for eliminating or reducing symptoms at the end of treatment for cellulitis (105). The recurrence rate of erysipelas has not been systematically determined for these latter antibiotics.

### Current Recommendations for Preventing Erysipelas Recurrence

Recommendations for preventing erysipelas recurrence have been addressed in several national guidelines (35). They are mainly based on observational studies with low evidence levels. Eliminating predisposing factors is considered mandatory in all of them. Initiating antibiotic prophylaxis is usually advised after the second episode. In patients with recurrent cellulitis, prophylactic treatment with oral penicillin or erythromycin or intramuscular benzathine penicillin has been recommended for as long as the predisposing factors persist (22). Five controlled studies have evaluated the response to antibiotic prophylaxis using either penicillin or erythromycin (35). All studies observed a decreased incidence of relapses during prophylaxis. In the erythromycin cohort, no relapses were observed during 18 months of prophylaxis. Unfortunately, patients were not followed after ending erythromycin intake (61). The prophylactic effects of penicillin for risk of cellulitis recurrence were not sustained after stopping prophylaxis (26, 35). Accordingly, the challenge of preventing erysipelas recurrence has remained unresolved.

### Insights From Otolaryngology: Streptococcal Eradication in Pharyngeal Carrier States

The issue of eradication has been extensively addressed for pharyngeal carrier states and recurrent episodes of streptococcal sore throat that may be considered as a condition analogous to recurrent erysipelas. The experience from treating recurrent streptococcal pharyngitis may therefore guide the directions for developing novel treatment strategies in recurrent erysipelas.

Oral penicillin V is the treatment of choice of GAS pharyngitis (104). Although most patients improve clinically within the first few days of treatment, oral antibiotic therapy requires 10 days for achieving maximum pharyngeal GAS clearance (111). Similar to erysipelas, however, penicillin failed to eradicate GAS from the throat in up to 35% of patients (112). Several trials have assessed eradication of streptococcal carrier states following treatment failures. They determined eradication rates following treatment with penicillin alone or in combination with rifampicin, cephalosporins, amoxicillin-clavulanate, macrolides, and clindamycin. Eradication rates for pharyngeal streptococcal carriage were higher in patients treated with clindamycin or amoxicillin-clavulanate compared to penicillin V treatment (113–115). Moreover, several studies confirmed superiority of cephalosporins over penicillins in GAS eradication (116–118). First-generation cephalosporins (such as cephalexin and cefadroxil) are considered alternatives to penicillin, especially in treatment failure or beta-lactam hypersensitivity (111, 119). A 5-day-course of azithromycin has also been reported to reach a 95% of GAS eradication (120). The efficacy of erythromycin is controversially discussed because of an increasing prevalence of erythromycin resistant GAS strains (121–123). Overall, the studies from otolaryngology propose that combining penicillin with clindamycin has the highest potential for eradication of pharyngeal streptococcal carrier states (124). Combination of penicillin with rifampicin may also be effective (125). Major trials for pharyngeal eradication are given in Table 1.

**Table 1:** Pharyngeal eradication rate of GAS.

**Studies**	**Agent**	**Route**	**Dosage**	**Duration**	**Eradication rate**
Morita et al. (120)	Azithromycin	Oral	12 mg/kg/day	5 days	95%
Orrling et al. (113)	Clindamycin vs. Penicillin V	Oral Oral	6.5 mg/kg b.i.d. (children) 300 mg t.i.d. (adults) 12.5 mg/kg b.i.d. for	10 days 10 days	100% 36%
Tanz et al. (114)	Clindamycin vs. Benzathine penicillin plus Rifampicin	Oral Intramuscular Oral	20 mg/kg t.i.d 600,000 units for < 27 kg 1,200,000 units for > 27 kg 20 mg/kg/d in 2 doses (max. = 600 mg/d)	10 days 10 days 4 days	92% 55%
Kaplan et al. (115)	Penicillin V vs. Amoxicillin-clavulanic acid	Oral Oral	Penicillin V 50 mg/kg/d in 4 doses 40 mg /kg t.i.d	10 days	31% 87%
Brook (118)	Cefdinir vs. Amoxicillin	Oral Oral	14 mg/kg/day or 600 mg once a day 40 mg/kg/day or 250 mg every 8 h)	10 days	92% 80%

### Antibiotic Combinations for Streptococcal Eradication in Erysipelas

Given the clinical experience with pharyngeal carrier states, attempts to eradicate streptococcal infection, and prevent erysipelas recurrence should primarily consider combination of antibiotics according to contraindications and potential adverse event risk in the individual patient. Clindamycin combined with penicillin is the first choice for the treatment of life-threatening GAS infections, such as necrotizing fasciitis, STSS, meningitis, and pneumonia. Clindamycin reaches sufficient bacteriostatic intracellular concentrations, inhibits production of streptococcal superantigens and other virulence factors, such as M protein, and combining penicillin with clindamycin was more efficient in treating deep infections such as GAS myositis than β-lactam antibiotics alone (126). Accordingly, such combinations were considered the most effective treatment for invasive *S. pyogenes* infection (127).

## The Future Handling of Erysipelas: Need for the Development of Novel Treatment Strategies Addressing Intracellular Streptococcal Reservoirs

Strategies addressing common erysipelas risk factors and reducing predisposing conditions, in particular curing inoculation sites for pathogens such as toe-web intertrigo, should remain standard clinical practice for preventing exogenous reinfection but apparently have limited effects on recurrence rates (61). It is further obvious from clinical trials and practical experience that current antibiotic regimens using β-lactam antibiotics can control acute GAS infections but are insufficient in preventing erysipelas recurrence (26, 35). Even long-term antibiotic prophylaxis does not reduce the recurrence rate in the post-prophylaxis periods (26, 35). Thus, current antibiotic regimens and measures targeting presumed risk factors do not address the actual causes of erysipelas recurrence and need to be reevaluated.

Insights into intracellular streptococcal uptake and persistence may now explain prevention failure. Penicillins, amoxicillin, and cephalosporins poorly penetrate cell membranes and do not reach sufficient intracellular bactericidal concentrations. Accordingly, intracellular streptococci are protected from β-lactam antibiotics (102). Bactericidal levels of penicillin in cell culture medium did not degrade intracellular streptococci while exposure of epithelial cells to either erythromycin or azithromycin could kill intracellular GAS efficiently in a cell culture model (103). Cephalothin and clindamycin were more effective in killing ingested GAS than was penicillin but less effective than erythromycin or azithromycin (103, 104). Such findings support that the hypothesis that the high incidence of erysipelas recurrence and failure of penicillin to prevent erysipelas relapses are indeed related to intracellular streptococcal uptake and a lack of intracellular penicillin activity.

### Development of Future Treatment Strategies

The implementation of novel approaches would benefit from additional preclinical and clinical antibiotic trials. These trials should assess antibiotics with intracellular efficacy on Gram-positive bacteria. The use of *in vitro* cell-culture infection models of intracellular streptococcal uptake and persistence might provide additional preclinical insights into the antibiotic efficacy of intracellular streptococcal eradication that can then be transferred into further clinical application (103).

The development of streptococcal vaccines might be another approach. Vaccination is effective in preventing infections or substantially improving the infectious disease course for various pathogens. M proteins have been considered major candidates for a GAS vaccine. Streptococcal M protein-specific antibodies promote opsonisation and subsequent streptococcal clearance by phagocytosis (128). M protein-specific antibodies are thought to represent the basis for antibody-mediated immunity from GAS infection, however, they protect only against GAS organisms of the same M serotype (129). An alternative approach for M protein-related vaccine development is therefore based on epitopes present in the C-repeat region toward the carboxy terminal of the M protein which are conserved among all or most GAS strains. M proteins, however, may induce cross-reactive immune responses against various human proteins which are thought to promote streptococcal sequelae including acute rheumatic fever, poststreptococcal glomerulonephirits, pediatric autoimmune neuropsychatric disorders associated with streptococcal infections (PANDAS) (17, 18, 99), and possibly also psoriasis (130). Former studies suggested that an M-type specific GAS vaccine may even predispose recipients to developing acute rheumatic fever rather than protecting from subsequent GAS infections (131). It will therefore be necessary to seek antigens that have minimal chance of inducing autoimmune sequelae. Various M protein and Non-M protein based streptococcal candidate vaccines are currently in preclinical or preliminary clinical development for the prevention of post-streptococcal diseases (132). A potential protective effect on erysipelas recurrence is currently speculative.

### Predicting the Risk of Erysipelas Recurrence in the Individual Patient

Tests for analyzing streptococcal persistence and predicting the risk of erysipelas relapses would be desirable and could help to decide the need for further preventive measure or regimens in the individual patient. Measurement of streptococci-specific antibodies such as anti-streptolysin O and anti-DNase B (133) in serum is useful as an indicator of a recent streptococcal infection and may indicate streptococcal persistence (99, 134). The specificity and sensitivity of serological tests to assess streptococcal persistence and predicting erysipelas relapses after a prior erysipelas episode has not been analyzed in detail. Culturing streptococci from acute erysipelas has been successful only in the minority of cases and is likely not meaningful after healing. Cultivation-independent molecular techniques based on the small subunit ribosomal RNA (16S rRNA) gene from tissue samples or blood (135) could be a more promising approach to determine the remaining presence of streptococci. They would allow for taxonomic differentiation, but the application of such molecular techniques is limited by the cost of Sanger sequencing and would be reserved for special indications and institutions. Alternative, PCR-based diagnostic approaches might be used to identify streptococcal DNA in tissue (136) but this would require biopsies from skin formerly affected by erysipelas. Thus, a first erysipelas episode is currently the most reliable predictor of relapse and will likely remain the actual indication for considering preventive measures.

### Considerations Regarding Intracellular Streptococcal Persistence for Clinical Practice

Intracellular persistence of streptococci may be a major cause of the yet unexplained high rate of erysipelas recurrence. Therefore, in the treatment of erysipelas special consideration should be given to eliminating possible intracellular streptococcal reservoirs. This aspect is relevant for two indications: (1) Treating first erysipelas episodes and (2) Treating erysipelas relapses. The treatment goals for both indications are basically identical: Control of acute infection and prevention of subsequent episodes. This would require antibiotics with sufficient intracellular activity. They include clindamycin, macrolides, and rifampicin. Combining clindamycin or rifampicin with penicillin is the currently most efficacious treatment of GAS infections. As long as no additional information from preclinical analyses and clinical trials on streptococcal persistence and eradication in erysipelas are available these antibiotic combinations should be considered as first-line treatment for preventing erysipelas recurrence, either by treating already first erysipelas episodes or—at least—the first episode of erysipelas recurrence. Additional costs and adverse event risks from the combination of these antibiotics in clinical practice might be compensated by reducing the incidence of erysipelas relapses and avoiding the high health costs and personal impairment resulting from treatment of recurrent erysipelas and progressing lymphedema. Careful monitoring of the relapse rate will be essential to determine the efficacy of this approach.

## Author Contributions

FJ and JP performed the literature review and data assessment. JP provided clinical photographs. MR performed the experiments on intracellular streptococcal uptake and persistence and generated the photographs of Figure 4. FJ, MR, and JP collaborated in writing the manuscript.

### Conflict of Interest Statement

The authors declare that the research was conducted in the absence of any commercial or financial relationships that could be construed as a potential conflict of interest.
